# Generation of entanglement in quantum parametric oscillators using phase control

**DOI:** 10.1038/srep13152

**Published:** 2015-08-19

**Authors:** J. C. Gonzalez-Henao, E. Pugliese, S. Euzzor, S.F. Abdalah, R. Meucci, J. A. Roversi

**Affiliations:** 1 Instituto de Física “Gleb Wataghin”, Universidade Estadual de Campinas, Unicamp 13083-970, Campinas, São Paulo, Brazil; 2 Istituto Nazionale di Ottica-CNR Largo E. Fermi 6, 50125 Firenze, Italy; 3 Dipartimento di Scienze della Terra - Università degli Studi di Firenze Via G. La Pira 4, Firenze, Italy

## Abstract

The control of quantum entanglement in systems in contact with environment plays an important role in information processing, cryptography and quantum computing. However, interactions with the environment, even when very weak, entail decoherence in the system with consequent loss of entanglement. Here we consider a system of two coupled oscillators in contact with a common heat bath and with a time dependent oscillation frequency. The possibility to control the entanglement of the oscillators by means of an external sinusoidal perturbation applied to the oscillation frequency has been theoretically explored. We demonstrate that the oscillators become entangled exactly in the region where the classical counterpart is unstable, otherwise when the classical system is stable, entanglement is not possible. Therefore, we can control the entanglement swapping from stable to unstable regions by adjusting amplitude and phase of our external controller. We also show that the entanglement rate is approximately proportional to the real part of the Floquet coefficient of the classical counterpart of the oscillators. Our results have the intriguing peculiarity of manipulating quantum information operating on a classical system.

Entaglement is one of the most important resources for several quantum information applications, for example, quantum cryptography, quantum metrology and quantum computation^1^,^2^,^3^. Unfortunately, for open systems the decoherence, caused by coupling with environment, degrades the quantum coherence and determines the disappearance of quantum behavior. The losses of entanglement are critical when they occur for relatively short times, i.e., for intervals less than the typical coherence time of the system^2^. Recently, coupled oscillators with variable frequency have been implemented to study the entanglement in simple quantum systems with a small number of excitations. An example is the one considered in ref. 4 where a photonic crystal interacting with a surface acoustic wave has been investigated. Other examples where the oscillators may exhibit time-dependent frequencies are given by optomechanical systems. In ref. 5 an oscillator with a spring constant amplitude modulated by a cosine function is described. In optomechanics^6^ and electromechanics^7^ systems it is also possible to find not constant oscillation frequencies. In ref. 8 it has been shown that using a periodic modulation it is possible to have small values of quantum entanglement, but only in the low temperature limit.

In the case of parametric coupled oscillators, it has been demonstrated that for a time dependent coupling it is possible to preserve entanglement even at high temperatures^9^. Such a result has been recently generalised to a non - Markovian regime showing that entanglement is allowed at higher temperatures with larger coupling strength to the baths and at smaller driving rates^10^. Later, it has been shown that the generation of entanglement depends on the classical dynamical stability of a free parametric oscillator^11^. The connection between entanglement and classical instability can be a powerful tool to investigate more complex systems, since the classical treatment can quickly reveal the parameter ranges in which the system will be entangled.

In this paper, in order to understand how two completely different features such as dynamical instability and entanglement are connected, we introduce a sinusoidal perturbation to the oscillation frequency of a parametric oscillator by a suitable phase modulation, i.e., *ω*(*t*) → *f*(Ω*t* + *ϕ*), where the phase *ϕ* is the control parameter. By varying amplitude and phase of the perturbation we show the correspondence between the classical regions of instability and entanglement.

## Results

### Dynamics of the oscillators

Our system consists of two coupled harmonic oscillators, 1 and 2, with time dependent frequencies in presence of a common environment. The Hamiltonian for this system is given by:


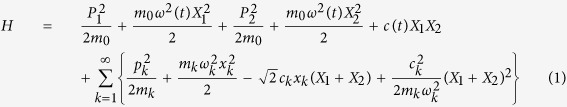


where *ω*(*t*) is the oscillator frequency of interest and {*X*_1_, *X*_2_, *P*_1_, *P*_2_} are the position and momentum operators for 1 and 2; *ω*_*k*_ and {*x*_*k*_, *p*_*k*_} are the frequencies and the position and momentum operators of the environment oscillators; the constants *c*_*k*_ correspond to coupling coefficients between the oscillators 1–2 and the environment and *c*(*t*) is the coupling between the oscillators. Introducing the operator transformations


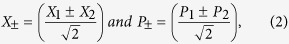


and after some algebraic manipulations we can write the Hamiltonian in the form:


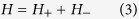



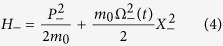



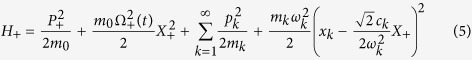


where 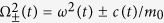.

*H*_−_ and *H*_+_ are respectively the Hamiltonian of a free oscillator and the Hamiltonian of the oscillator coupled with the environment.

The analysis of the entanglement of this system was performed by determining the evolution of the position and momentum operators of both oscillators and evaluating the covariance matrix elements in the phase space *R* = (*X*_1_, *X*_2_, *P*_1_, *P*_2_), given by


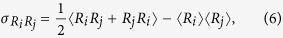


where *i* and *j* vary from 1 to 4.

The covariance matrix is more easily obtained by using the operators {*X*_+_, *X*_−_, *P*_+_, *P*_−_}, in fact the Hamiltonian *H*_+_ and *H*_−_ commute among themselves and this allows an independent analysis. In our case, without loss of generality, we consider the initial state of the system as the tensor product of coherent states 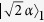 and 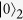, that on the basis of operators {*X*_+_, *X*_−_, *P*_+_, *P*_−_} are 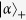 and 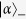.

### Covariance elements of the system {X_−_, P_−_}

The evaluation of the average values of the operators 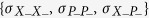 can be obtained using the Heisenberg representation^12^. Making the substitution 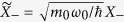, 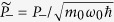 and 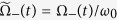 the coordinates of the system become dimensionless and the calculation of the evolution of covariance matrix elements are performed through the following system of differential equations (where the tilde was omitted to simplify the notation):


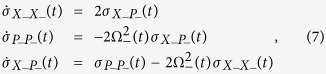


whose solution is given by:


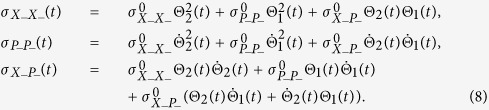


where 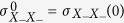, 
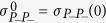
 and 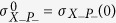. The functions Θ_1_(*t*) and Θ_2_(*t*) are the solutions of the differential equation:


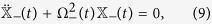


where Θ_1_(*t*) and Θ_2_(*t*) are evaluated considering the initial conditions Θ_1_(0) = 0, 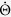
_1_(0) = 1, and Θ_2_(0) = 1 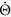
_2_(0) = 0, respectively.

### Covariance elements of the system {X_+_, P_+_}

The presence of the environment operators {*x*_*k*_, *p*_*k*_} in the Hamiltonian (5) requires an accurate analysis of the operators {*X*_+_, *P*_+_} in comparison with the free harmonic oscillator (4). To perform this analysis we introduce the following dimensionless coordinates:


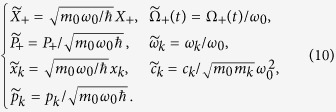


For the sake of simplicity hereafter the tilde symbol will be omitted. The solution of this system of equations was obtained by the Feynman-Vernon path integral formalism (See ref. 13 for more details about the density operator). We present here the basic ideas to calculate the Covariance Matrix elements.

This formalism helps us to determine the time evolution of the density matrix. We define the density matrix as the time and position coordinate function


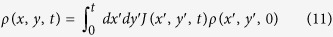


where the matrix 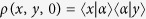 and the propagator *J*(*x*, *y*, *t*), in the ohmic case^13^, is


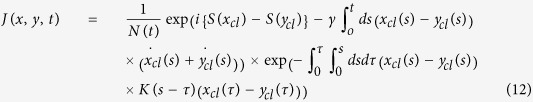


where the kernel function 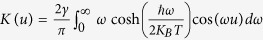. The function *S*(.) is the action calculated from the Lagrangian:


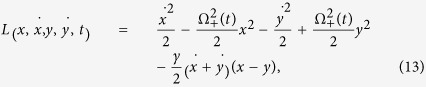


which is obtained from the classical version of Hamiltonian (5), with the coordinate *x* playing the classic role of the quantum position operator *X*_+_. Finally the functions *x*_*cl*_(*t*) e *y*_*cl*_(*t*) are the classical paths calculated from the Euler-Lagrange equation^14^ given by the Lagrangian (13):


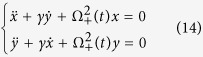


The solution of the system (14) can be determined making the transformations 
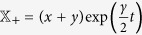
 and 
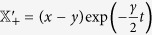
 obtaining:


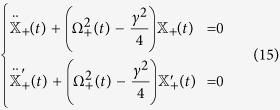


where the initial conditions for *x*_*cl*_(*t*) and *y*_*cl*_(*t*) are given by *x*_*cl*_(0) = *x*′, *y*_*cl*_(0) = *y*′, *x*_*cl*_(*t*_*f*_) = *x*_*f*_ and *y*_*cl*_(*t*_*f*_) = *y*_*f*_. Once obtained the *ρ*(*x*_*f*_, *y*_*f*_, *t*_*f*_) function is possible to find any covariance matrix elements of a generic observable *Â* through the expression 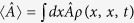.

### Stability of the oscillators

The connection between classical instabilities and the existence of quantum entanglement relies on the elements of the covariance matrix of the operators {*X*_1_, *X*_2_, *P*_1_, *P*_2_} which depend, as stated before, on the solutions of the following differential equations:


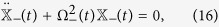



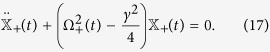


The differential equations (16) and (17) are the classical counterparts corresponding to the Hamiltonians *H*_−_ and *H*_+_ respectively. In ref. 11, it was shown that entanglement of parametric oscillators 1 and 2 strongly depends on the stability of the solutions of Eq. (16) which is associated with the position and momentum operators in the Hamiltonian *H*_−_. It only occurs for values for which the oscillator “−” is unstable. On the other hand in ref. 9 it was shown that any coupled oscillator interacting with a different reservoir has to satisfy the inequality 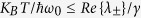 in order to become entangled. Note that in ref. 9 the Floquet coefficient is *iμ*_*M*_ = *λ*_±_, then 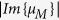 = 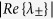, where *λ*_±_ are the Floquet coefficients^15^ associated with Eqs. (17) and (16), respectively. Differently from ref. 9, the oscillator “−” is not directly coupled to the thermal reservoir. This is due to the nature of coupling between the operators *X*_1,2_ with the reservoir and the particular coordinate transformation *X*_±_ which makes the operator *X*_−_ corresponding to a free oscillator. This implies that the entanglement condition occurs when the oscillator “−” is unstable or the condition 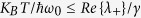 is satisfied by the oscillator “+”. Indeed, we have found that, for low temperature and small values of dissipation rate, the “+” oscillator is unstable and this condition allows entanglement, a result not yet reported in the literature. Since we are interested in systems at high temperatures, the entanglement will be achieved only when the oscillator “−” is unstable.

In order to obtain entanglement control, the oscillator frequency is perturbed as follows


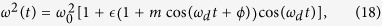


where *ω*_*d*_ is the external driving frequency, *m* and *ϕ* are the amplitude and the phase of the external perturbation respectively. With this assumption we can rewrite Ω_−_ in an adimensional form as


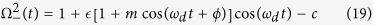


where the coupling term takes the form 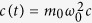.

**Figure 1:**
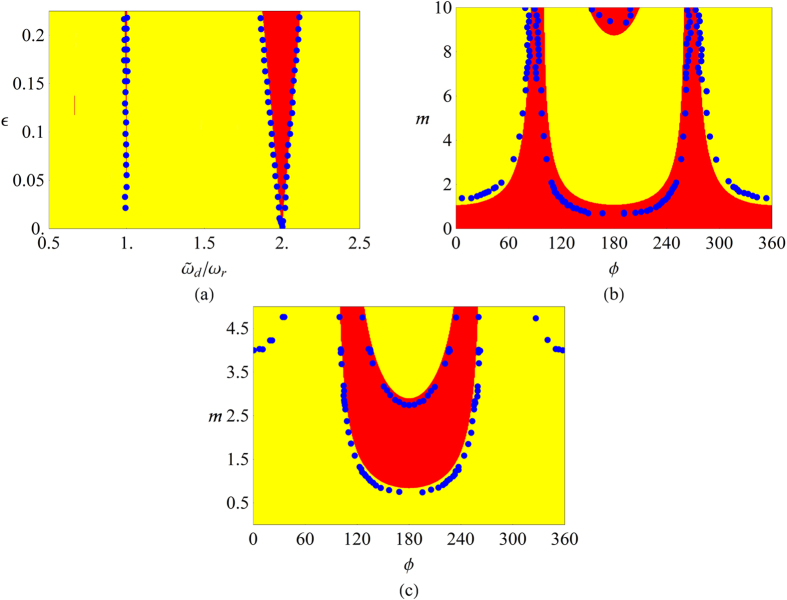
Stability maps of Eq. (16). The red and yellow colors represent the stable and unstable behavior respectively. Blue dots are the stability borders as experimentally measured. In (**a**) *m* = 0, in (**b**) *ω*_*d*_/*ω*_0_ = 2*ω*_*r*_ and in (**c**) *ω*_*d*_/*ω*_0_ = 1.77*ω*_*r*_.

Eq.(16) represents a periodic differential equation due to the structure of the frequency function *ω*(*t*), and it can be solved applying the Floquet coefficient theory^16^. In this framework we first consider *m* = 0 to investigate stable and unstable behavior. The stability map is shown in Fig. 1(a) where 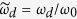. To apply the phase control technique we set two values of *ω*_*d*_, one in the stable and the other one in the unstable region, with a fixed value of 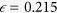. The dynamical behavior of the oscillator is modified as shown in Fig. 1(b,c) where stability and instability regions are reported in the parameter space *m* and *ϕ*. These figures are obtained considering *c* = 0.09 and defining a new auxiliary variable 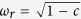.

**Figure 2:**
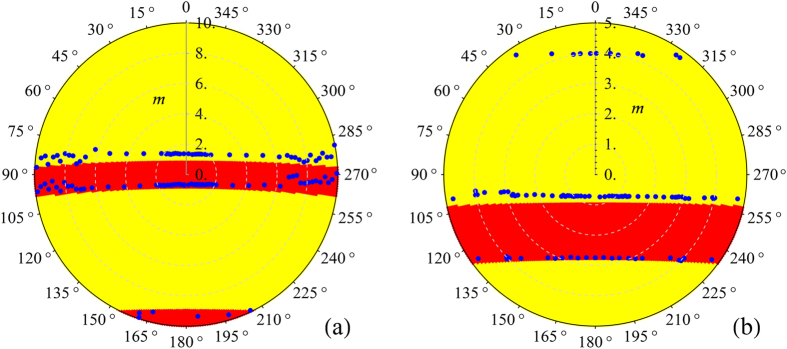
Polar map representation. (**a**) corresponds to Fig. 1(b) and (b) to Fig. 1(c) respectively.

The system described by Eq. (16) has been experimentally investigated implementing its analog electronic version. The presence of diverging solutions, associated with unstable behavior, was detected by observing the saturation regime in the output signals in the electronic circuit. The borders of the stability regions were consequently determined (see blue dots in Fig. 1). The stability maps in Fig. 1(b,c) are also represented by using (see Fig. 2) polar coordinates, defined as *m*_*x*_ = *m* sin*ϕ*, *m*_*y*_ = *m* cos*ϕ* where the radial coordinate is now the variable *m*. In this new representation the regions of instabilities are approximately given by horizontal bands.

In Fig. 2(a) the instability of the oscillator “−” occurs in two regions approximately defined by −0.615 ± 0.037 ≤ *m* cos*ϕ* ≤ 1.199 ± 0.044 and −9.198 ± 0.057 ≤ *m* cos*ϕ*. In Fig. 2(b) the main instability region occurs at −2.762 ± 0.005 ≤ *m* cos*ϕ* ≤ −0.705 ± 0.004. In the upper part of this figure unstable points approximately described by the line *m* cos*ϕ* = 3.922 ± 0.019 are also reported. The intriguing relationship existing between the classical instability obtained from the Floquet coefficients and the existence of quantum entanglement, for exactly the same parameter values, will be investigated in the next section.

## Discussion

### Entanglement

In this section we will show how it is possible to control the entanglement by manipulating the parameters *m* and *ϕ*. This is achieved by looking at the quantum correlation between the two oscillators. Noting that the final system state is a Gaussian one^17^, the theorem of positive partial transpose (*PPT*)^18^ can be used as a criterion for entanglement. Thus, to quantify entanglement we use the logarithmic negativity defined as:


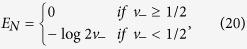


where the value of *v*_−_ is given by:


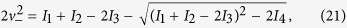


where 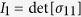, 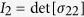, 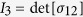 and 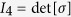, the matrices *σ*_11_, *σ*_22_ and *σ*_12_ being sub-blocks of the covariance matrix *σ* given by


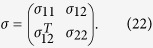


If *E*_*N*_ ≠ 0 the system is entangled and when *E*_*N*_ = 0 the oscillators 1 and 2 are separable. Although the logarithmic negativity is not a concave or convex function, it is a monotonous function of entanglement since, in average, it doesn’t increase under Local Operations and Classical Communication (*LOCC*) operations or operators that conserves *PPT*^19^,^20^.

**Figure 3:**
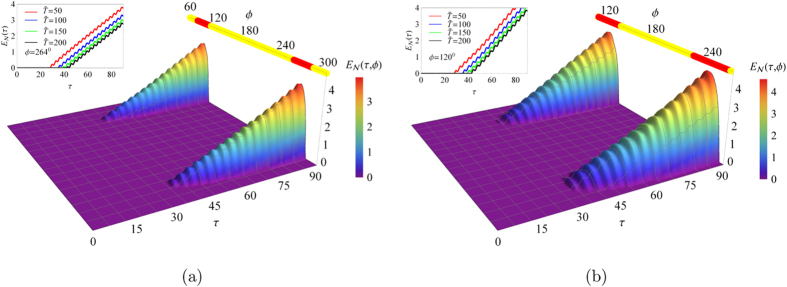
Surfaces of logarithmic negativity *E*_*N*_(*τ*, *ϕ*) and curves of *E*_*N*_(*τ*). In (**a**) *ω*_*d*_/*ω*_0_ = 2*ω*_*r*_, *m* = 4; in the inset *ϕ* = 264°. In (**b**) *ω*_*d*_/*ω*_0_ = 1.77*ω*_*r*_, *m* = 4; in the inset *ϕ* = 120°. In both insets the used values are: *γ* = 0.01*ω*_0_, *c* = 0.09, 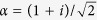, 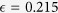 and 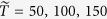 and 200.

In Fig. 3, for *m* = 4, we plot the logarithmic negativity as a function of time *τ* (*τ* *=* *ω*_0_*t*) and phase *ϕ* at given values of the environment temperature 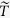 (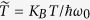) for both stability maps shown in Fig.1(b,c). For 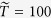, on all mentioned surfaces was added a top bar, where the yellow or red colors correspond to *ϕ* values where we have a stable and unstable behaviour in the solutions of Eq. (16) (oscillator “−”), respectively.

From Fig. 3, it is observed that entanglement only occurs for those values of classical parameters, *m* and *ϕ*, where the oscillator “−” is unstable. In this way, we can generate or suppress quantum entanglement controlling the classical parameters. We can also observe that entanglement does not occur instantaneously but it is necessary to wait for a certain interval of time for the oscillators to be entangled. Such a delay time *τ*_*R*_ can be obtained from Eq. (22) and from the relationship det(*σ*_12_) ≤ 0 which defines the entanglement condition^21^. This fact is in agreement with the results shown in Fig. 3(a) where there is no entanglement for 0° < *ϕ* < 60° and 300° < *ϕ* < 360°, and with those of Fig. 3(b) for 0° < *ϕ* < 80° and 280° < *ϕ* < 360°. After reaching the minimum time *τ*_*R*_ needed to achieve an entangled state, the logarithmic negativity *E*_*N*_(*τ*,*ϕ*) shows an approximately linear behavior with small variations as shown in the inserts on the left side of the Fig. 3(a,b), for *ϕ* = 264° and *ϕ* = 120*°* respectively. From the inserts it can be seen that an increase in the temperature produces a decreasing in the entanglement and an increasing in the time necessary to generate it, as expected. But these two concomitant behaviors do not change the average rate *ξ* of increase of the logarithmic negativity.

**Figure 4:**
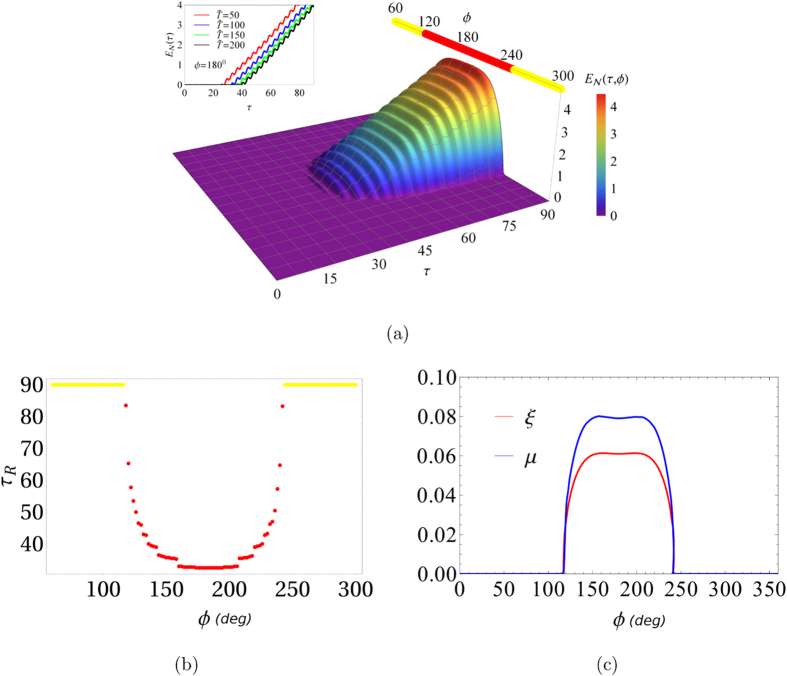
Surface of logarithmic negativity and relationship between entanglement rate and Floquet coefficient. (**a**) Surface of logarithmic negativity *E*_*N*_(*τ*,*ϕ*) for *ω*_*d*_/*ω*_0_ = 1.77*ω*_*r*_ and *m* = 2; in the inset *ϕ* = 180°. (**b**) entanglement start time *τ*_*R*_ vs *ϕ* and (**c**) entanglement rate *ξ* and Floquet coefficient *μ* vs phase *ϕ*.

In Fig. 4(a) we plot the logarithmic negativity *E*_*N*_(*τ*, *ϕ*) for the same parameter values corresponding to the stability map of Fig. 1(c), with *m* = 2.0, *γ* = 1×10^−2^ω_0_, *c* = 0.09 and 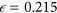. In this map, only one unstable region occurs for 116.8° < *ϕ* < 243.2°. It can be seen from this figure that the system displays quantum entanglement only for values of *ϕ* for which the oscillator “−” is unstable. The time and temperature dependencies are similar to those reported in Fig. 3.

The behavior of *τ*_*R*_ as a function of the phase *ϕ*, is reported in Fig. 4(b) for the same parameter values used in Fig. 4(a). In this figure the red dots indicate values of angular coordinate *ϕ* for which the oscillator “−” changes its behavior from stable to unstable (yellow dots for the opposite behavior). For *ϕ* values where the oscillator “−” is stable no entanglement is found, meaning that the values of *τ*_*R*_ shown in yellow are actually lower than the threshold values. In Fig. 4(c), the entanglement rate *ξ* (blue curve) and the real part of the Floquet coefficient *Re*{*λ*_−_} = *μ* (red curve) are reported as a function of the phase *ϕ*. The curves *ξ*(*ϕ*) and *μ*(*ϕ*) have similar behaviors. A linear fit of *ξ*(*ϕ*) versus *μ*(*ϕ*), i.e., *ξ*(*ϕ*) = *aμ*(*ϕ*)*−b* leads to the values *a* = 1.480 ± 0.018 and *b* = 0.010 ± 0.001, respectively. This implies that the rate of entanglement is linearly related with the real part of the Floquet coefficient (at least where this is greater than zero). On the other hand, the time *τ*_*R*_ required for the onset of entanglement depends on *μ*^−1^. This is due to the logarithmic behavior of a linear function of time and the *μ* dependence on the average dissipation rate. This last conclusion results from the fact that *E*_*N*_(*τ*) ≈ *ξ*(*ϕ*)*τ* − *E*_0_(*ϕ*) (where *E*_0_(*ϕ*) is simply a constant). The relationship between *τ*_*R*_ and *ϕ*, and the knowledge of Ω_−_(*t*) allows us to determine from the classical instabilities the starting time of entanglement and also its increase rate. In order to summarize, during the swap between stable and unstable regions, we need a minimum time *τ*_*R*_ approximately given by *τ*_*R*_ ≈ *E*_0_(*ϕ*)/(*aμ*(*ϕ*) − *b*) to reach an entangled state.

## Conclusions

In this work we analyze the generation and extinction of quantum entanglement due to phase control. The model is based on two coupled quantum parametric oscillators with a time dependent oscillation frequency interacting with a reservoir. The regions of classical instabilities have been numerically and experimentally investigated confirming that they are associated with positive values of the logarithmic negativity. Moreover, even with a time dependent frequency, the logarithmic negativity shows an approximately linear behavior with small fluctuations. Furthermore, we observed that entanglement states require a time *τ*_*R*_ to start. Entanglement is only observed at high temperatures, if one of the classical oscillators exhibits unstable behavior. As a consequence, phase control is an useful tool for its creation and extinction. We also observe that the entanglement increase rate is approximately proportional to the real part of the Floquet coefficient of the classical oscillator “−”. This means that is possible manipulate entanglement and its increase rate recurring to the classical counterpart.

## Methods

As already mentioned, the system described by Eq. (16) has been implemented by an analog electronic circuit. The circuit has been realized by using commercial electronic components. The harmonic oscillator with natural angular frequency *ω*_*r*_, has been obtained by using a cascade of four operational amplifiers LT1114CN by Linear Technology. A function generator Hameg HM 8131-2 provides the sinusoidal driving signal 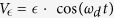 and a multiplier chip MLT04G by Analog Devices implements the product 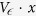 (where 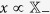). Experimental evidence of diverging solutions was given by observing the saturation imposed by the integrated electronic components. By varying *ω*_*d*_, the system approaches the instability regions showing a decreased amplitude modulation at the angular frequency difference *ω*_*d*_−*ω*_*r*_. Signal clipping is observed when saturation is reached. Phase control has been realized including two additional multipliers MLT04G and a second function generator Hameg HM 8131-2 which provides the modulation signal *V*_*m*_(*ϕ*) = *m*·*cos*(*ω*_*d*_*t* + *ϕ*). The two function generators have been connected in a master-slave configuration ensuring a given and adjustable value of their phase difference. The behavior of the controlled system depends on the strength *m* and the phase difference *ϕ* between the two signals *V*_*m*_(*ϕ*) and 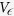. In particular, at *ϕ* = 180° (see Fig. 1(b)), the system changes from unstable to stable behavior and vice versa increasing *m*. Similarly, in Fig.1(c), the opposite transition is observed. The experimental data have been acquired by means of a TDS 7104 Tektronix digital oscilloscope.

## Additional Information

**How to cite this article**: Gonzalez-Henao, J. C. *et al.* Generation of entanglement in quantum parametric oscillators using phase control. *Sci. Rep.* **5**, 13152; doi: 10.1038/srep13152 (2015).
